# Excessive Atmospheric Pressure the Cause of Cholera
*De la Nature, du Traitement, et des Préservatifs du Choléra. Par F. X. Poznanski, Docteur en Médecine de l'Académie de Wilno. pp. 51. Paris: Bailliére et Fils. 1857.


**Published:** 1857-10

**Authors:** 


EXCESSIVE ATMOSPHERIC PRESSURE THE CAUSE OF CHOLERA.*

Dr. Poznanski believes that in the circulation of the blood two forces are concerned—an active, arising from the circulatory organs; and a resistent force, consisting in atmospheric pressure. The centrifugal force, or normal circulation, of the blood, he states to be the result of the due adjustment of these forces. In cholera, the balance is deranged, he argues, by an excess of the atmospheric pressure. In support of this view, he adduces, with other arguments, the following. Cholera is endemic in countries subject to excessive atmospheric pressure, as the East Indies, South Carolina, and other similar regions. Epidemics of cholera have always been preceded and accompanied by an excess of atmospheric pressure; and their intensity has been in proportion to the excess. There may be much truth in what Dr. Poznanski says; but we should imagine him to be, like many others, a man of one idea.

Several other works lie on our table, but we can only mention them. The Directors of James Murray's Royal Asylum for Lunatics, near Perth, have issued their *Thirtieth Annual Report.* This asylum, it will be recollected, is conducted on the most enlightened principles. Mr. H. W. Lobb publishes a pamphlet on a preparation of cow's milk partly deprived of casein, so as to fit it for the use of infants. He therefore gives it the euphonious title of *Mincasea (minus casei).* It may be very good; but the affair looks too commercial. Dr. Hyde Salter's *Introductory Address,* delivered at the opening of the Twenty-third Session of the King's College Medical Society, in October 1856, contains some good arguments in favour of combination among students for mutual professional improvement. We have received *Sanitary Reports* from Messrs. Nicholas, Whiteman, Godrich, Pearce, and Drs. Conway Evans and Septimus Gibbon. They contain much valuable matter. Preparations are being made for a complete *resumé* of all the documents.

* De la Nature, du Traitement, et des Préservatifs du Choléra. Par F. X. POZNANSKI, Docteur en Médecine de l'Académie de Wilno. pp. 51. Paris: Baillière et Fils. 1857.

